# Empowering Undergraduates to Fight Climate Change with Soil Microbes

**DOI:** 10.1089/dna.2021.0551

**Published:** 2022-01-12

**Authors:** Elias Taylor-Cornejo

**Affiliations:** 1. Department of Biology, Randolph-Macon College, Ashland, Virginia, USA.

**Keywords:** microbial fuel cell, electrogenic bacteria, distance learning, microbiology education

## Abstract

The burning of fossil fuels to meet a growing demand for energy has created a climate crisis that threatens Earth's fragile ecosystems. While most undergraduate students are familiar with solar and wind energy as sustainable alternatives to fossil fuels, many are not aware of a climate solution right beneath their feet—soil-dwelling microbes! Microbial fuel cells (MFCs) harness energy from the metabolic activity of microbes in the soil to generate electricity. Recently, the coronavirus disease 2019 (COVID-19) pandemic transformed the traditional microbiology teaching laboratory into take-home laboratory kits and online modes of delivery, which could accommodate distance learning. This laboratory exercise combined both virtual laboratory simulations and a commercially available MFC kit to challenge undergraduate students to apply fundamental principles in microbiology to real-world climate solutions.

## Introduction

The summer before I began graduate school in the Department of Plant and Microbial Biology at UC Berkeley, I volunteered at a local greenhouse in my hometown of Denver, Colorado. This greenhouse was run by a nonprofit organization, called The GrowHaus, which sought to increase access to fresh, healthy food in a community that had been deemed a “food desert” (Dutko *et al.*, 2012). At the GrowHaus, I helped run a free summer program for high schoolers in the community, where we taught the basics of healthy nutrition and sustainable urban agriculture. I taught a lesson on the biological principles of composting, including the important role that soil microbes play in recycling nutrients like carbon and nitrogen in the environment (Rousk and Bengtson, 2014).

At a community event at the end of summer, one of my students gave a presentation that explained how composting is an easy and inexpensive way to make nutrient rich soil to start a backyard garden and how the majority of what we consider “trash” is actually compostable. I realized that by making science relevant to his everyday life, this student was motivated to address a real problem in their community. While I was fortunate to learn microbiology in a university laboratory equipped with microscopes, incubators, and reagents to culture microbes in a sterile environment, the students at the GrowHaus were learning microbiology in a what was once an abandoned flower shop. Despite limited resources, the students learned fundamental concepts in microbial diversity, metabolism, and growth using nothing but food scraps, plant litter, and a handful of soil.

Fast forward to the Fall of 2020, I began my first semester as an Assistant Professor of Microbiology at Randolph-Macon College in Ashland, VA. I was eager to launch my research program and design biology courses to inspire the next generation of scientists to become drivers of scientific innovation to solve real-world issues impacting human health and the environment. However, with the coronavirus disease 2019 (COVID-19) pandemic in full swing, like many educators, I was obligated to rethink my mode of instruction to fit a virtual format. For my Introduction to Microbiology course, this meant transforming the traditional microbiology teaching laboratory into virtual simulations and take-home laboratory kits (Herzog and Mawn, 2020). A major aspect of my teaching philosophy is to engage students to apply the subject matter to solve real-world problems.

However, how was I going to fit an entire semester worth of microbiology teaching laboratories into a small box that I could mail to students? I needed a laboratory that was safe, multipurpose, and relevant to the student's everyday lives. Much like my days at the GrowHaus, I found myself needing to teach microbiology without the tools and equipment of a traditional microbiology laboratory. I had a “light bulb” moment—by displaying how electricity can be harnessed from the metabolic activity of microbes in the soil, students could see how fundamental principles of microbiology are being applied to a renewable energy technology to address the threat of climate change.

With a few handfuls of soil, a plastic container, and a few pieces of low-cost electrical circuit hardware, bacteria in the soil can generate power in a device called a microbial fuel cell (MFC) (Jude and Jude, 2015). The MFC laboratory became the primary hands-on research experience for the students to use throughout the semester to learn about microbial diversity, metabolism, growth, and its application in biotechnology.

## Climate Change and the Need for Renewable Energy

When you flip on a light switch or charge your smartphone, how often do you stop and think where this energy comes from? The vast majority of the electricity produced globally comes from burning fossil fuels such as coal, oil, and natural gas, which are limited, nonrenewable resources (International Energy Agency, 2020). Burning fossil fuels produces carbon dioxide, which is the primary contributor to global greenhouse gas (GHG) emissions (Olivier *et al.*, 2005). GHG emissions from human activity has greatly contributed to global warming, which has sparked an international response, most notably under the 2015 Paris Climate Agreement, which advocated for measures that would prevent global temperatures from rising more than 1.5°C above preindustrial levels (Schleussner *et al.*, 2016).

Reversing the impact of human-induced climate change is an international effort that requires a drastic reduction in global GHG emissions by keeping fossil fuels in the ground and switching to alternative, renewable energy sources (Rogelj *et al.*, 2015). Regrettably, roughly 80% of electricity in the United States is still being produced from nonrenewable energy sources (U.S. Energy Information Administration, 2021). Although renewable energy technologies such as wind energy, hydroelectric energy, and solar energy all come with their caveats in regard to implementation and efficiency, the combination of using many different renewable energy sources offers a viable alternative to replacing nonrenewable energy (Hansen *et al.*, 2019).

**FIG. 1.**
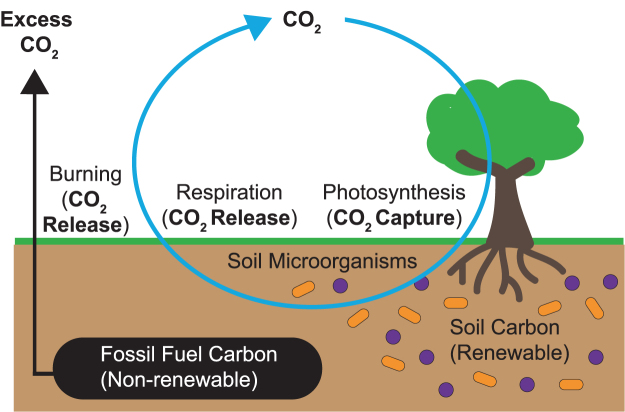
Relative atmospheric carbon dioxide contribution from nonrenewable versus renewable carbon sources. Burning fossil fuels releases carbon dioxide from nonrenewable resources resulting in excess carbon dioxide in the atmosphere. Carbon dioxide that is captured by photosynthesis serves as a renewable carbon source that fuels cellular respiration of living organisms, including soil microorganisms (*purple spheres* and *orange rods*). The capture and release of carbon dioxide by photosynthesis and cellular respiration, respectively, represent a balanced carbon cycle.

Atmospheric carbon is balanced by two competing metabolic activities, photosynthesis and cellular respiration (Tkemaladze and Makhashvili, 2016). Photosynthesis removes carbon dioxide from the atmosphere and converts it to glucose. On the other hand, cellular respiration consumes glucose for energy and releases carbon dioxide in the process. MFCs represent a renewable energy source because microbes are consuming organic biomass in the soil that was created recently, opposed to burning fossil fuels that releases carbon dioxide into the environment that was captured during ancient photosynthetic events (Dunaj *et al.*, 2012; Javed *et al.*, 2018). Although the metabolic activity of soil microbes can produce carbon dioxide, this carbon dioxide continues to be recycled among the current organism on Earth (Fig. 1).

## Electrogenic Bacteria Produce Electricity

Living cells are specialized to perform biochemical reactions that extract energy from biological molecules, which powers their own essential cellular processes. One such process is cellular respiration—a metabolic process that uses the movement of electrons to generate a form of energy that the cell can use, called adenosine triphosphate (ATP) (Lecomte *et al.*, 2018). During cellular respiration in bacteria, electrons are stripped away from an electron donor, relayed through an electron transport chain in the cell membrane, and deposited onto a terminal electron acceptor. Ultimately, this produces electrochemical gradient across the cell membrane and a proton motive force that drives the formation of ATP through an enzyme complex called ATP synthase.

Microbial cells have adapted to use a wide range of electron donors and electron acceptors for cellular respiration. In aerobic environments, where oxygen is plentiful, oxygen is a common terminal electron acceptor. However, in anaerobic environments, where oxygen is not available, electrons can be deposited onto a variety of terminal electron acceptors, including molecules like nitrate or sulfate (Lecomte *et al.*, 2018).

While the name cellular respiration implies that this occurs inside of cells, there are many examples of microbes that dump their electrons onto terminal electron acceptors that are outside of the cell. For example, some bacteria dump their electrons onto insoluble metals in the environment (e.g., Fe^3+^ or Mn^4+^) (Gralnick and Newman, 2007). Bacteria that donate their electrons to materials that are outside of the cell are considered electrogenic, because they have the capability of producing electricity (Lovley, 2012). Extracellular electron transfer by bacteria is an abundant source of electrons in nature, which is being harnessed to produce renewable energy using MFC technology (Logan *et al.*, 2019).

## A Microbiology Laboratory Suitable for Distance Learning

I strive to develop a sense of agency in students, that through learning microbiology, they can improve technologies that address real-world problems. In light of the need to accommodate distance learning during the COVID-19 pandemic, I had students safely build an MFC at home or in their dorm room. The MFC was used throughout the semester to demonstrate how fundamental principles of microbiology can be applied to confront the threat of climate change. Students were provided with a commercially available MFC kit, which contained all of the hardware needed to construct an electrical circuit, and three cups of garden soil from the local hardware store (Magical Microbes, 2021).

**FIG. 2.**
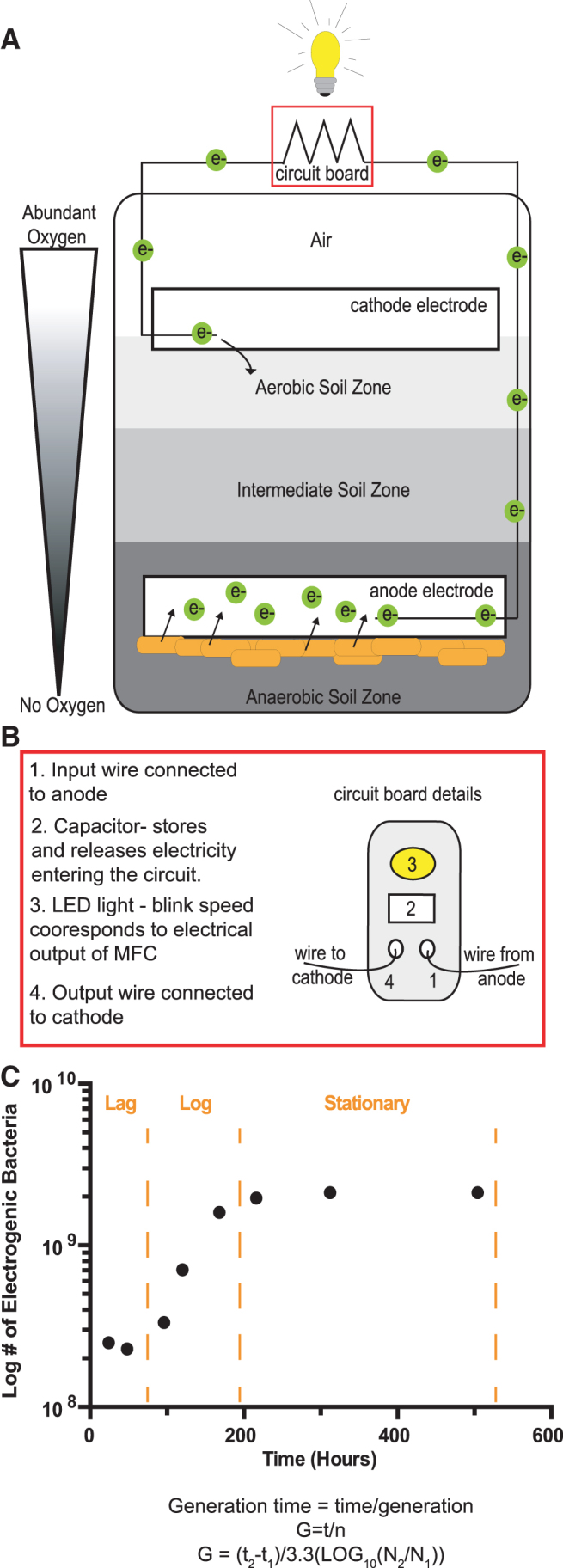
An MFC cell is powered by electrogenic bacteria. **(A)** MFC design. An oxygen gradient is established in a container with soil and two electrodes. Oxygen is most abundant at the surface of the soil that is exposed to air (aerobic soil zone), then is gradually depleted deeper into the soil, until eventually no oxygen is present (anaerobic soil zone). Electrogenic bacteria (*orange*) transfer electrons to the electrode that is placed in the anaerobic soil zone. The movement of electrons (*green*, e-) toward the cathode creates an electric current between the two electrodes and provides the electricity needed to power a device (light bulb) through the circuit board (*red box*). **(B)** Detailed electrical connections and components of the MFC circuit board (anode connection, cathode connection, capacitor, and LED light). **(C)** Example MFC data. Growth curve generated from converting power output measurement to number of electrogenic bacteria. Relative boundaries of canonical growth phases for bacterial populations are indicated (lag, log, and stationary phase; *orange dotted lines*). Generation time of the electrogenic population is calculated using two data points from log phase using the equation generation time = time/generation (G = t/n), which is expanded into G = (t_2_−t_1_)/3.3(LOG_10_(N_2_/N_1_)). LED, light-emitting diode; LOG, logarithm; MFC, microbial fuel cell.

The kit also included a light-emitting diode (LED) light that blinks when the MFC produces a sufficient electrical current. Before constructing their MFC, students learned the principles of electricity and practiced building simple circuits using virtual laboratory simulation (Labster, 2021). The MFC is constructed in layers. One electrode is placed deep in the soil, in the anaerobic zone that supports the growth of electrogenic bacteria; the other electrode is placed on top of the soil, where there is plenty of oxygen to serve as an electron sink at the end of the circuit (Fig. 2A).

To construct an electrical circuit, a wire is embedded in the anode and connected to a circuit board that houses the LED light and a small capacitor that stores and pulses the electricity that is generated from the MFC (Fig. 2B). Another wire is connected from the circuit board to the cathode to complete the circuit. Electrons flow from the anode, to the capacitor, to the LED light, and to the cathode and are ultimately deposited abiotically onto oxygen at the surface of the wet soil to generate water through an oxygen reduction reaction (Ma *et al.*, 2019) (Fig. 2A).

Virtual laboratory simulations complemented the hands-on MFC laboratory kit to demonstrate fundamental principles of microbiology and laboratory techniques that would typically be taught in person. For example, in a typical, in-person introductory microbiology laboratory, students learn how to isolate microbes from environmental samples using sterile technique, and how to quantitatively measure growth of microbes in pure culture. In the absence of an in-person microbiology laboratory, students learned these fundamental techniques using virtual laboratory simulations, but then generated their own dataset with their MFCs (Labster, 2021; Magical Microbes 2021).

Data were collected using a smartphone application that records the power output of their MFCs based on blink speed, which directly correlates to the number of bacteria that are depositing electrons onto the electrode (El-Naggar *et al.*, 2010; McLean *et al.*, 2010). In other words, the electric current and power output will increase as the bacterial population grows and as more bacteria are donating electrons to the electrode.

During the remote learning environment of the COVID-19 pandemic, students were able to use their MFC to measure bacterial growth without any of the standard tools that are available in a traditional microbiology laboratory, such as a spectrophotometer or mountains of nutrient agar plates, and could even observe the typical growth phases of bacterial populations (Fig. 2C) (Buchanan, 1918; Ben-David and Davidson, 2014). Furthermore, they were able to use these measurements to calculate the generation time, which is an important skill that allows scientists to make direct comparison of how bacterial populations respond to different treatments or variables (Supplementary Figs. S1, S2). Therefore, in the absence of an in-person microbiology laboratory, students were able to learn valuable, translatable, skills for future careers in microbiology.

Another common introductory microbiology laboratory is for students to construct a self-contained microbial soil community called a Winogradsky column, which illustrates the interdependent microbial metabolisms that recycle important nutrients such as carbon, sulfur, and nitrogen (Parks, 2015). Students first performed virtual laboratory simulation from the HHMI Biointeractive “Winogradsky Column: Microbial Ecology in a Bottle” to understand how aerobic and anaerobic environments are established and how microbes can be classified based on their reliance on certain carbon and energy sources (i.e., autotroph, heterotroph, chemotroph, and phototroph) (HHMI BioInteractive, 2021).

Building an MFC reinforced the concepts from the virtual laboratory because this self-contained vessel of mud essentially mimics the vertically stratified metabolic niches that are found in a Winogradsky column. For example, if constructed properly, the MFC will be aerobic at the top of the vessel and anaerobic at the bottom of the vessel.

During instruction, students are introduced to the redox tower and how a molecule's reduction potential equates to its ability to accept electrons (Seager *et al.*, 2012). The high reduction potential of oxygen makes it a favorable terminal electron acceptor; however, in anerobic environments, microbes use alternative electron acceptors, such as the carbon-based material (graphite) of the anode (Magical Microbes, 2021).

The MFC also was used to illustrate the important role that microbes play in recycling of nutrients in soil by connecting the MFC to the microbial process of composting. Students were encouraged to add compostable household materials that would normally be discarded to serve as soil supplements, such as paper (carbon source), coffee grounds (nitrogen source), or boiled egg yolk (sulfur source) (Nimni *et al.*, 2007; Liu and Price, 2011). With everyone constructing their MFC laboratory remotely, there were too many confounding variables to conduct a properly controlled experiment to establish cause and effect; however, students were able to compare the power output of their MFCs and make connections to their virtual Winogradsky lesson by discussing how nutrient composition of soil supports the growth of certain types of microbes.

When students first assemble their MFC, many believe that it “does not work” because the LED light on their MFC does not start blinking immediately. It typically takes a few days for the voltage to reach the minimum 0.35 V needed to activate the circuit board (Magical Microbes, 2021). During this time, I encourage students to think about where electricity comes from, and they begin to realize that they are “making” electricity opposed to just plugging into an outlet to consume electricity. In reality, electrogenic microbes are the ones “making” electricity by depositing electrons onto the anode, but the students are establishing proper anaerobic conditions for electrogenic microbes to thrive in the MFC, to harness electricity as electrons travel to the cathode.

Unlike, traditional “cookbook” style laboratories, where students follow a protocol and achieve expected results, inquiry-based laboratories that recreate an authentic research experience, which includes trial and error, offer many benefits in student learning, critical thinking, and research skills (Lord and Orkwiszewski, 2006; Gormally *et al.*, 2009). Like most real-world laboratory experiments, an MFC requires a bit of optimization to establish conditions that are just right to support microbial growth. For some students, their MFCs did not blink even after a week of waiting for the voltage to build up, in which case, we troubleshooted the construction and assembly of the MFC in our virtual sessions. In most cases, air pockets were visible in the soil near the anode.

With some prompting, students realized that having oxygen at the anode would interfere with the anaerobic respiration that we were trying to achieve. Regardless, the MFC laboratory challenges students to think critically about how to create an ecosystem for microbes to thrive and they must wrestle with fundamental concepts in microbiology such as microbial diversity, metabolism, and growth to get their MFC to work. Therefore, in addition to the fundamental laboratory skills and quantitative analysis, the MFC laboratory offered a meaningful problem-solving opportunity that connected the learning objectives from class to a hands-on laboratory activity.

## Future Innovators for Confronting Climate Change

As we begin to return to in-person instruction, the lessons learned from investigating the microbial world in a few cups of soil will power on! My long-term goal is to design an interdisciplinary, classroom undergraduate research experience in which students in the Biology department collaborate with peers in other departments (i.e., Physics, Chemistry, and Engineering) to design a more efficient MFC (Shortlidge and Brownell, 2016). For example, students can test the impact that variables such as soil composition, temperature, or different materials have on the power output of the MFC (Dunaj *et al.*, 2012; Jude and Jude, 2015).

Sustainable, low-cost MFCs have been designed for use in developing countries, where unreliable access energy can hinder simple tasks, such as powering a lamp or charging a cell phone (Jain, 2011; Acosta-Coll *et al.*, 2021). Overall, the goal of the MFC laboratory was to get students thinking about alternative ways that energy can be produced, particularly for this climate-challenged generation. Most of all, by being forced out of the traditional microbiology laboratory during the COVID-19 pandemic, it enabled us all to see that microbiology is not just about growing bacteria on Petri dishes, but instead, it is knowing that there is an abundance of diverse microbes that can be found in just a few cups of soil, and those microbes just might save the planet.

## Author's Contribution

E.T.-C. (corresponding author) was the sole contributor to the preparation of all figures, drafts, edits, and final version of this article.

## Supplementary Material

Supplemental data

Supplemental data
