# Supplementary material for: Empowering Undergraduates to Fight Climate Change with Soil Microbes
Source: DNA Cell Biol. 2022 Jan 12;41(1):58–63. doi: 10.1089/dna.2021.0551 (PMC8787709; doi:10.1089/dna.2021.0551)
Supplement: Supplemental data [file Supp_FigS1.pdf]

POWER (μWATTS)	ESTIMATED # OF ELECTROGENIC BACTERIA	TIME (HOURS)
11	2.3×10^8	0
12	2.5×10^8	24
11	2.3×10^8	48
16	3.3×10^8	96
34	7.1×10^8	120
77	1.6×10^9	168
94	2.0×10^9	216
102	2.1×10^9	312
102	2.1×10^9	504

Generation time (G) = time(t) / generation (N)

$G = t/n$

$G = (T_1 - T_2) / 3.3 (\text{Log}_{10} (N_2/N_1))$

$G = (120 – 96) / 3.3 (\text{Log}_{10} (7.1 \times 10^8 / 3.3 \times 10^8))$

$G = 24 / 3.3 (\text{Log}_{10} (2.1))$

$G = 24 / 3.3 (0.33)$

$G = 24 / 1.1$

G = 21.8 generations per hour

Supplemental Figure 1. Example data from MFC with generation time calculation. Example MFC data. Estimated number of electrogenic bacteria using provided MudWatt calculator. Generation time calculated using example MFC data with 96 and 120 hour timepoints.
