# Supplementary figures and images for: Empowering Undergraduates to Fight Climate Change with Soil Microbes
Source: DNA Cell Biol. 2022 Jan 12;41(1):58–63. doi: 10.1089/dna.2021.0551 (PMC8787709; doi:10.1089/dna.2021.0551)

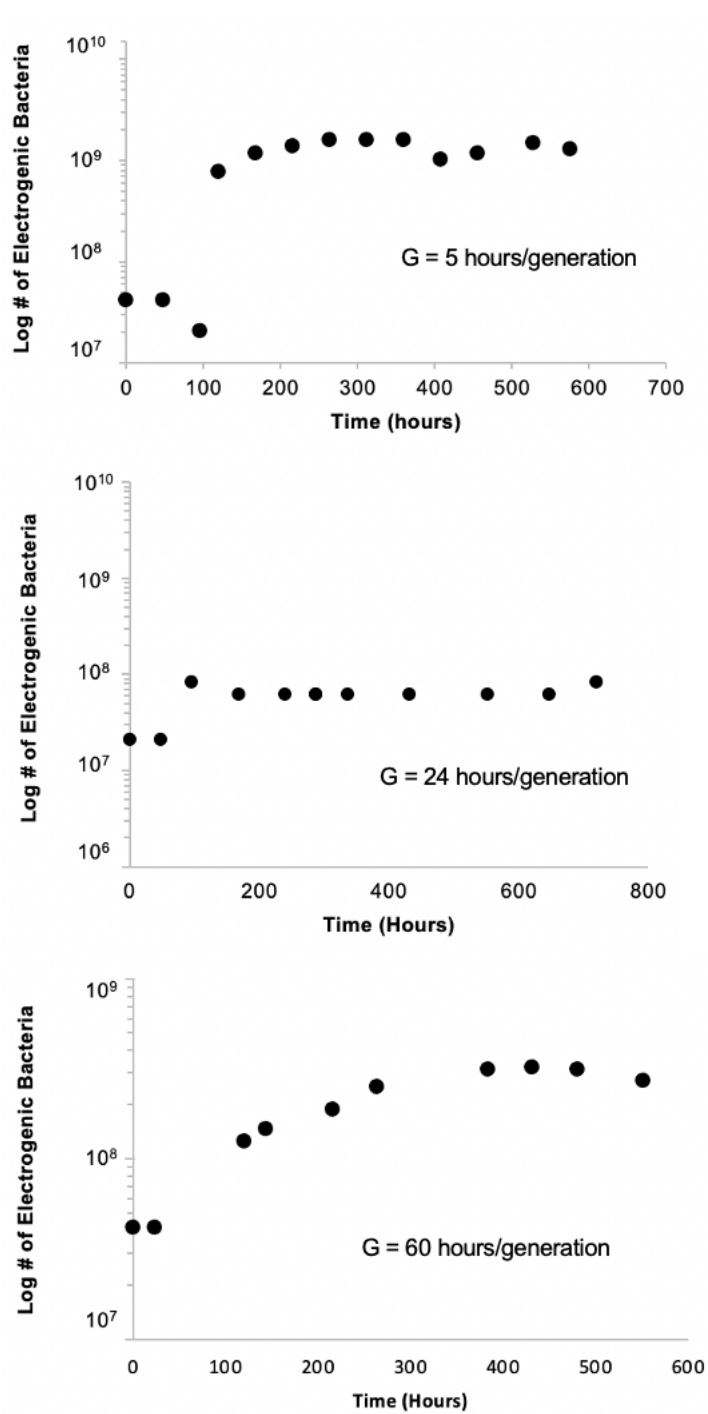

Supplemental Figure 2. Sample student MFC data with generation time (G) calculation.

Supplement: Supplemental data [file Supp_FigS2.pdf]
